# Socio-Economic Development and Mental Health: Case Study of the Spanish Region of Aragon (2010–20)

**DOI:** 10.3389/fpsyg.2022.899278

**Published:** 2022-06-10

**Authors:** Carmen Bentué-Martínez, Marcos Rodrigues, Rafael García-Foncillas López, José María Llorente González, María Zúñiga-Antón

**Affiliations:** ^1^Department of Geography and Territorial Planning, University of Zaragoza, Zaragoza, Spain; ^2^Department of Microbiology, Pediatrics, Radiology and Public Health, University of Zaragoza, Zaragoza, Spain; ^3^Aragon Health Research Institute, Zaragoza, Spain; ^4^Aragon Health Service, Zaragoza, Spain

**Keywords:** primary health care, depression, social determinants of health, territorial development, decision making, socioeconomic status

## Abstract

**Introduction:**

Considering health as a cross-cutting element of all public policies leads to rethinking its interactions with the environment in which people live. The collection of large volumes of data by public administrations offers the opportunity to monitor and analyze the possible associations between health and territory. The increase in the incidence and prevalence of mental health diseases, particularly depression, justifies the need to develop studies that seek to identify links with the socioeconomic and environmental setting.

**Objective:**

The objective of this study is to explain the behavior of the depression in a mediterranean region of Northeastern Spain from an ecological and diachronic perspective.

**Methods:**

We conducted a correlation and multivariate logistic regression analysis to identify explanatory factors of the prevalence of depression in 2010 and 2020 and in the variation rate. Potential explanatory factors are related to the socioeconomic status and to the territorial development level.

**Results:**

The regression models retained both socioeconomic and territorial development variables as predictors of the prevalence in both years and in the variation rate. Rural areas seem to play a protective role against the prevalence.

**Conclusion:**

It is under the territorial prism that epidemiological studies could offer useful guidelines for proactive decision-making. The integration of data on diseases and territory must be considered when developing policies for the creation of healthier environments and for directing health services with more specific resources to where they may be needed.

## Introduction

Advancing upon our current understanding of “health” raises the need to consider it beyond the mere lack of discomfort including the overall wellbeing of the individual as well as social welfare (Santoro-Lamelas, 2016). Mental health conditions, and specifically depression-related ones, are the main cause of disability in western countries (WHO, 2017). The increasing provision of services linked to economic growth is often associated to wellbeing and, in parallel, good health. Indeed, economic development has fostered medical advances and increasing access to health services, which ultimately contribute to welfare (Jorm and Ryan, 2014). However, economic growth does not necessarily improve mental health and several studies support the notion that psychiatric disorders and depression go hand in hand with industrialization and urbanization, as it favors income inequality (Gupta et al., 2016; Yu and Wang, 2016; Ribeiro et al., 2017). There is a growing interest in monitoring and reducing inequalities both from the approach of the UN SDGs and the OECD, from the assertion that inequality affects the social fabric and harms long-term economic growth (OECD, 2015; United Nations, 2015). The drivers of depression are not yet fully understood, being it attributed to different factors such as genetic predisposition, personality traits, as well as impaired social interactions (Dunn et al., 2015; Gariépy et al., 2016; Cai et al., 2020). The socioeconomic status, gender or the residential environment could also pose a risk of depression (Freeman et al., 2016; Kuehner, 2017; Razzak et al., 2019). Other risk factors could be specifically manifested in rural or urban settings, especially in terms of diagnosis, treatment and use of health services (Wang, 2004; Fortney et al., 2010; Purtle et al., 2019).

The Determinants of Health (DH) approach and the Health in All Policies [HiAP; (WHO, 2014)], stress the importance of policies, actions and interventions outside the direct remits of the healthcare sector, e.g., regional planning or economic development (Puska, 2007; Ollila, 2011). These approaches advocate for assessing the wide array of protective and/or risk factors mediating health conditions, i.e., individual lifestyles, social and community networks, food production systems, the socioeconomic and residential environment, the access to health services or policies in place (Dahlgren and Whitehead, 2007; Gnanapragasam et al., 2021). Parallel to the inception and evolution of the HiAP and DH approaches, the “information revolution” has enabled compiling and cataloging large amounts of data with increasing complexity in their structures and interrelationships (the so-called Big Data). Today, public administrations collect large amounts of data on a regular basis that can be put at the service of healthcare and resource management.

We hypothesize that an uneven territorial development and the environmental settings influence health status and, specifically, depression and mental conditions. Hence, landscape and urban planning may play a role in improving mental health. It is in this context of necessary interaction between health, environment, and data availability to satisfy the pressing need for management that this study is proposed. We investigate the link between socioeconomic and territorial indicators, and the prevalence of depression in Aragon. We leverage official data sources to conduct a diachronic analysis of their association. The socioeconomic factors are expressed through deprivation indicators referring to the demographic structure, the work environment, and the level of education. Those referring to the territorial development illustrate the accessibility to facilities and services and to the transportation network, the condition of residential buildings and the quality of the landscape. The hypothesis of this study is that a greater vulnerability to the prevalence of depression corresponds to a higher socioeconomic deprivation and lower levels of territorial development.

## Materials and Methods

The methodological process was divided into three phases. First, a spatial database was generated on depression prevalence (as dependent variable) and indicators on socioeconomic environment and territorial development (as independent variables). Second, the variables were aggregated at the Basic Health Area (BHA) level to prepare the cartographic representation and statistical analysis. Third, a correlation and logistic regression analysis was performed to explore associations between the variables.

### Study Population and Sample Size

The target population of our study are all individuals inside the National Health System registered in Aragon (Spain) during 2010 or 2020. The sample consists of patients with a digital medical record being 18 years and older, including elderly people (over 65), having been diagnosed with Depression, according to the International Classification in Primary Care (ICPC) of the World Organization of Family Doctors (WONCA) (ICPC 2 – P76). to the universal nature of the health system and the absence of other health care data providers, the sample analyzed in this study can be considered as representative of practically the 100% of the adult population diagnosed with depression.

### Study Area

**FIGURE 1:**
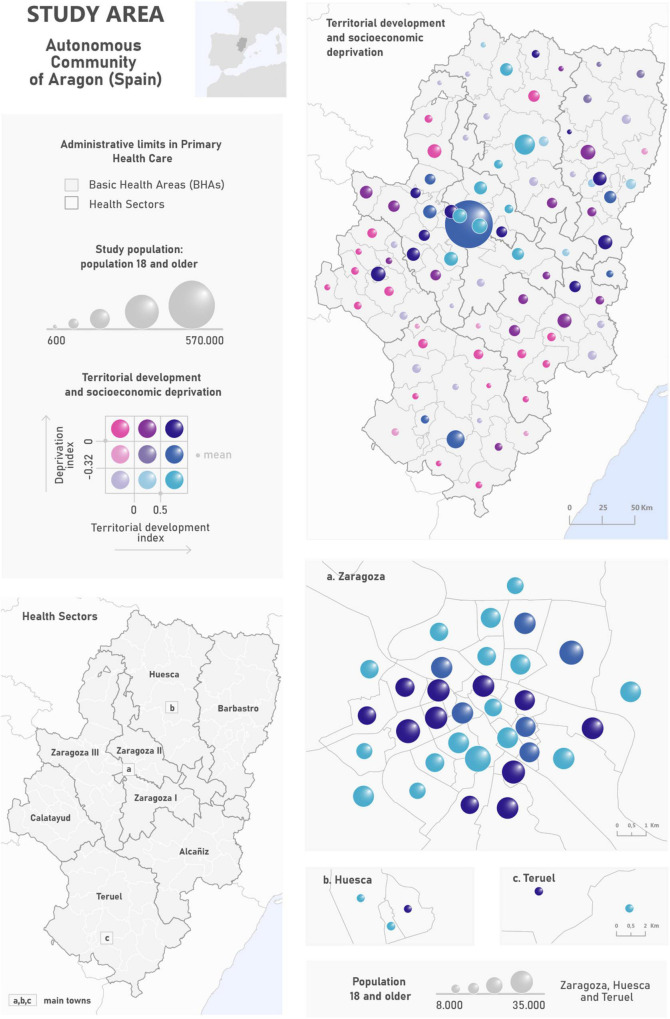
Study area and spatial distribution of the privation and territorial development indexes by BHA.

The study area is the Autonomous Community of Aragon. The territorial administrative unit for primary health care in Aragon is the BHA. BHAs are groupings of municipalities except in the case of the three main towns, Zaragoza, Huesca and Teruel, which group census tracts. BHAs are grouped into eight health sectors: Huesca, Barbastro, Zaragoza I, Zaragoza II, Zaragoza III, Calatayud, Teruel, and Alcañiz. The region covers an area of 47.720 square km with a population density of 27.8 inhabitants per square km (in 2021). The multivariable map in Figure 1 illustrates the large contrasts that characterize this region in terms of population distribution, territorial development, and socioeconomic conditions. We find the main population concentrations in the middle and mid-west of the region. These areas are characterized by a high level of territorial development that in many cases, is accompanied by a high socioeconomic deprivation (dark cyan tones). The BHAs displayed in purple illustrate intermediate territorial development. These regions host health-care centers assigned to people in the surrounding settlements. Populations living in these areas are even young or aged and the deprivation conditions can be high (dark purple tones). In contrast with previous ones, we found numerous BHAs that show a smaller population size, especially in the north and south of the region. These are characterized by lower levels of territorial development and an overaged demographic structure. These disadvantages can be accompanied by socioeconomic deprivation (intense magenta tones). These contrasts in the settlement distribution are derived from physical factors such as the arrangement of the main relief units which ultimately explain climate conditions and land cover. There is a gradient from wet and cool conditions in the two surrounding mountain ranges (Pyrenees, 3,400 m.a.s.l. and Iberian System, 2,300 m.a.s.l.) to warm and dry situations in the central corridor of the River Ebro Valley.

### Variables and Data Collection

The dependent variable of this study is the prevalence rate of depression. The Aragon Health Sciences Institute of the University of Zaragoza provided standardized rates by gender and age at BHA level in the years 2010 and 2020. As independent variables, we retrieved and compiled a comprehensive set of socioeconomic and demographic indicators from official datasets and databases in Aragon (Table 1).

**TABLE 1:** Variables of the study, indicators, and units of measure.

Dependent variable	Unit of measure
Prevalence of depression in 2010 and 2020 by BHA.	Percentage
**Independent variables**	
**Socioeconomic factors**	
*Deprivation index*. Linear combination of Unemployment, Casual Wage Earners, Insufficient Instruction in persons aged 16 to 64 and Foreigners.	Range from −2.33 to 3.04
*Temporary workers*: ratio between (a, as numerator) the number of temporary or temporary employees and (b, as denominator) the employed population.	Percentage
*Manual workers*: ratio between (a) persons aged 16 and over employed in catering, personal services, protection, salesclerks, skilled workers in agriculture and fishing, craftsmen, skilled workers in manufacturing, construction and mining, except plant and machinery operators, plant and machinery operators, assemblers and unskilled workers; and the employed population.	Percentage
*Unemployment*: ratio between (a) unemployed persons seeking their first job or who have previously worked and the employed and (b) unemployed persons either seeking their first job or who have previously worked.	Percentage
*Low education level*: ratio between (a) illiterate population or having incomplete primary education and (b) the population 16 years and older.	Percentage
*Low education level in young population*: ratio between (a) 16–29 years old illiterate population or having incomplete primary education and (b) the population 16 years and older.	Percentage
*Low education level in foreign population*: ratio between (a) foreign illiterate population or having incomplete primary education and (b) the population 16 years and older.	Percentage
*Overaging rate*: ratio between (a) people aged 65 years and older and (b) total population.	Percentage
*Population 85 and older*: ratio between (a) people aged 85 years and older and (b) total population.	Percentage
*Single-person > 65 households*: ratio between (a) households consisting of an only person aged 65 years and older and (b) total households.	Percentage
*Households without internet access*: ratio between (a) households without internet access and (b) total households.	Percentage
**Territorial development factors**	
*SITD*: synthetic index constructed by the combination of partial indicators related to Economy, Households, Facilities and services, Mobility and Vital scenery.	Range from −0.8 to 12
*Economy*: represents economic activity, considering general economic indicators such as the number of Social Security affiliations, unemployment, youth unemployment, and sectorial variables on primary, secondary, or tertiary sector activities. Also incorporates a set of demographic variables that indirectly condition economic development such as population growth, distribution and demographic structure by sex and age.	Range from −1.2 to 17
*Residential environment*: illustrates building characteristics using indicators such as the age, the cadastral value, and the state of conservation.	Range from −0.7 to 14.1
*Facilities and services*: the criteria of accessibility to facilities and services is measured in time through the road network to different educational, health and administrative facilities.	Range from −2.2 to 6.6
*Mobility*: the mobility factor relates to ease of transportation, access to the high-capacity road and the rail network and communication technologies.	Range from −1.1 to 4.6
*Landscape*: takes in account related to landscape such as its quality and social appreciation, as well as other indicators such as the percentage of the municipal surface included in figures of environmental protection and the presence of trails that run through the municipality.	Range from −0.5 to 7.2

The core information for our analyses comes from the Aragon’s Deprivation index (Compés Dea et al., 2018). The database was created by the General Directorate of Public Health of the Government of Aragon, the Aragon Statistics Institute (ASI) and the Department of Microbiology, Preventive Medicine and Public Health of the University of Zaragoza. They developed a cross-sectional study linking mortality and deprivation, taking the BHA as the unit of analysis, aggregating data from all the inhabitants of Aragon as of January the 1st 2011 (Spanish Population Census of 2011). Out of the 26 indicators that make up the Deprivation index, we selected the following: unemployment, casual wage earners, insufficient instruction and Foreigners. In this work, we analyze both the Deprivation Index and a selection of the original indicators.

We included a second set of indicators related to territorial development, collected from the spatial database of the Synthetic Index of Territorial Development of Aragon (SITD). The SITD was created by the General Directorate of Territorial Integration, Mobility and Housing of the Government of Aragon. It consists of a combination of factors that illustrate different development axes: Economy, Residential environment, Facilities and services, Mobility and Landscape.

### Statistical Analysis

To analyze the association between the prevalence of depression and the socioeconomic and territorial variables, we calculated the Spearman’s or Person’s R correlation coefficient, retrieving both the coefficient value and its significance level (*p*-value). Pearson was calculated when data were normally distributed, while Spearman was used if at least one covariate did not follow a normal distribution. To further investigate potential relationships, we conducted a multivariate regression analysis. Since the assumption of normality does not hold for all covariates, we leaned toward logit regression. We constructed a separate binary response variable for 2010 and 2020, classifying as “1” all records above the median prevalence rate (6.37 in 2010 and 10.6 in 2020) and as “0” otherwise. Additionally, we analyzed the change in the prevalence rate, calculating the variation rate between 2010 and 2020. Again, the resulting variable was recoded as 0–1 using the median as separation threshold. Three separate models were evaluated (2010, 2020 and change). For each one, we calibrated a null model containing all variables as candidate predictors. We discarded those found to be collinear (VIF > 5), subsequently retaining significant predictors through a backwards stepwise procedure minimizing the AIC. Each “final” model was submitted to the Hosmer Lemeshow goodness of fit test and the Likelihood ratio test. Additionally, we computed the Nagelkerke pseudo-R2 coefficient to inform about the performance of the model.

## Results

**FIGURE 2:**
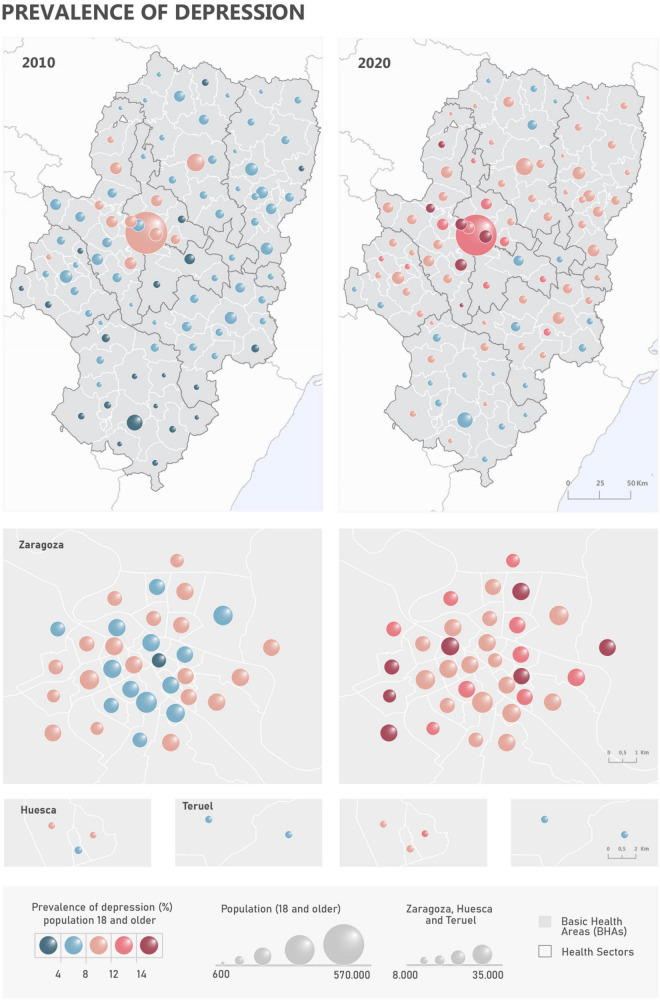
Spatial distribution of the prevalence of depression in 2010 and 2020.

In 2010, 80,030 adult individuals were diagnosed with depression in Aragon, a figure that rose to 123,174 in 2020, increasing by 53% during the period. Conversely, the total population in the same age group slightly decreased from 1,111,812 in 2010 to 1,096,878 in 2020. The highest prevalence rates in 2010 were observed around Zaragoza and its metropolitan area while the lowest were observed in the southern and northern ends of the region, especially in Teruel (Figure 2). All socioeconomic factors -aside from wage earners, unemployment, and the Deprivation index itself- attained significant and negative correlations in both 2010 and 2020. Territorial indicators showed stronger associations, being all significant and positively correlated but landscape quality, which aroused a negative correlation (Table 2). This implies that BHAs with an overaged population, low instruction level, which are less advantageous in terms of economic development and access to infrastructures/equipment are less prone to depression; meaning that rural enclaves may exert a sheltering effect. But the same indicators -either socioeconomic or territorial- were found significantly associated with the change in the prevalence rate between 2010 and 2020. However, the direction of the association was reversed in all cases. Hence, regardless of the suggested protective effect of rural areas, it is precisely these settings that are increasing faster in relative terms. The results from the logit regression models support the behavior from the correlation analyses (Table 3). The 2010 and 2020 models retained the same variables as predictors of the prevalence of depression, i.e., overaging rate, manual workers, and landscape quality. In both cases it means that a lower level of over-aging, a lower proportion of manual workers and a lower quality of the landscape are associated with an increased probability of depression. In the case of the variation rate the model identified three variables with explanatory capacity (though only one was significant *p* < 0.05): low level of education in the young and foreign population and the overaging rate. The direction of the relationship suggests a greater probability of finding a higher increase in prevalence by 2020 in areas with an overaged demographic structure accompanied -to a lesser extent- by a lower level of education in the foreign population. The results from the goodness of fit tests are favorable in all cases (HL > 0.05 and LR < 0.05). However, the performance of the models was higher in the case of 2010, followed by the 2020 and the variance models.

**TABLE 2:** Correlations between the prevalence of depression and the variables studied.

	Prevalence of depression		
	2010	2020	Variable
**Socioeconomic indicators**			
Deprivation index	−0.035	−0.093	−0.011
Temporary workers	0.074	0.074	−0.096
Manual workers	−0.476**	−0.396*	0.418*
Unemployment	0.113	0.069	−0.127
Low education level	−0.517**	−0.414**	0.448**
Low education level in young population	−0.195*	−0.195*	0.195*
Low education level in foreign population	−0.189*	−0.189*	0.117
Overaging rate	−0.551**	−0.551**	0.476**
Population 85 and older	−0.463**	−0.343**	0.406*
Single-person > 65 households	−0.398**	−0.296**	0.298**
Households without internet access	−0.571**	−0.427**	0.502**
**Territorial development indicators**			
SITD	0.622**	0.622**	−0.522**
Economy	0.597**	0.597**	−0.519**
Residential environment	0.560**	0.560**	−0.497**
Facilities and services	0.648**	0.648**	−0.496**
Mobility	0.603**	0.603**	−0.496**
Landscape	−0.615**	−0.615**	0.503**

*p-value < 0.05; **p-value < 0.001.

**TABLE 3:** Summary of logit regression models.

Model	Variable	Beta Coeff.	*p*-value	pseudo R2	HL test	LR test
2010	Overaging rate	−0.24	0.02*	0.56	0.56	0.00
	Manual workers	−0.07	0.00**			
	Landscape	−1.86	0.02*			
2020	Overaging rate	−0.17	0.03*	0.33	0.91	0.00
	Manual workers	−0.05	0.01**			
	Landscape	−0.71	0.06			
Var	Low education level in young population	0.06	0.14	0.28	0.47	0.02
	Low education level in foreign population	−0.03	0.15			
	Overaging rate	0.34	0.00**			

*p-value < 0.05; **p-value < 0.001; HL, Hosmer Lemeshow test; LR, Likelihood ratio.

## Discussion

In this study we explore the spatial distribution of the prevalence of depression from a socioeconomic and territorial approach. Our findings reinforce the notion that the circumstances in which people live are linked to the variability in the prevalence of depression (Lopizzo et al., 2015; Rautio et al., 2018). The rural-to-urban continuum seems to be the underlying driver of the spatial distribution of the prevalence rates. However, the observed associations and relationships revealed manifold nuances. The initial hypothesis about a greater vulnerability expected in areas with greater socioeconomic deprivation and lower levels of territorial development, can only be partially accepted.

The BHAs of the city of Zaragoza, its metropolitan area and those of the central-east sector of the region correspond to predominantly urban areas where high rates of depression have been observed in 2010 and 2020. The explanatory factors identified by the regression models (higher probability of prevalence with lower overaging, proportion of manual workers and landscape quality) lead to reconsideration of these areas. The positive correlations between the prevalence and the territorial development factors (except for the quality of the landscape) support such reexamination, leading us to qualify the initial hypothesis. We observe that an advantageous situation in terms of territorial development does not necessarily protect against the prevalence of depression. In urban areas, the social ties are less solid than in rural environments, the feeling of belonging to the community is lower, the urban way of life is associated with greater stress, and the enjoyment of natural spaces compared to rural areas is less affordable. In contrast, rural enclaves are usually located in natural environments attaining higher landscape quality than their urban counterparts. In addition, social relationships are strong and derive to a large extent from the existence of a sense of attachment to the place of residence. In this regard, the seemingly sheltering effect of rural livelihoods (Ayuso-Mateos et al., 2001) could be explained by the social support networks (Rubio-Aranda et al., 2012; Gariépy et al., 2016), the greater sense of belonging to the community (Kim et al., 2004; Romans et al., 2010) and the positive effects of rural landscapes on mental health (Velarde et al., 2007).

Regarding socioeconomic status (SES), there is a scientific consensus on the link between SES and mental health (Ostler et al., 2001; Patel et al., 2018). However, in the particular case of depression, a greater heterogeneity in the results and controversy exists due to the way the psychiatric disorder and the SES is measured as well as to the contextual background (Lorant et al., 2003). It is noteworthy that in our study the composite indicator of deprivation did not explain the prevalence of depression, while some partial index factors did. The explanatory factors of the variance model revealed a greater probability of an increase in the prevalence in overaged areas with lower educational attainment. This factor has been highlighted in previous studies for its protective role against the prevalence of depression (Zimmerman and Katon, 2005; Bjelland et al., 2008; Freeman et al., 2016), especially in the elderly (Miech and Shanahan, 2000; Domènech-Abella et al., 2018). In turn, other SES measurements such as the marital status or the income level have proven to be more significant than educational level (Inaba et al., 2005). In other studies, these associations also vary depending on population characteristics such as sex and age (Elliott, 2001; Back and Lee, 2011; Arias-de la Torre et al., 2018).

We consider that in addition to the educational attainment, there are other factors that may contribute to explain the increase in the prevalence of depression in rural areas in Aragon. The median prevalence of depression in Aragon was 6.37% in 2010 and 10.6% in 2020. The prevalence from primary care consultations offered by the PREDICT study was 12.2% (King et al., 2006) and 10.6% in the ESEMED-Spain project (Gabilondo et al., 2010). These figures reveal a possible under-diagnosis in the prevalence of depression in 2010 (Llorente et al., 2018), a frequent situation, especially in the elderly (Faisal-Cury et al., 2022). The regression results revealed an increased prevalence of depression in areas with aged population (Kyu-man et al., 2019; Linder, 2019). This implies that, although the elderly population living in rural areas may enjoy protective environmental factors against mental health conditions, they still deserve special attention from the healthcare system. In this context, it should be noted that between 2010 and 2020, the healthcare sector in Aragon has improved the mechanisms of diagnosis and the follow-up of the prevalence through the so-called “Aragon’s mental health plan 2017–2021.” This effort has been translated into a higher integration of mental health centers in the BHAs and the incorporation of the figure of the consultant psychiatrist for primary care. In addition, screening tests for geriatric depression have been incorporated into medical history follow-up programs, such as the Yesavage test (Yesavage, 1988). Therefore, the increase in prevalence in rural settings could be linked to the increased accuracy in the diagnosis capability.

In summary, this study illustrates an assessment based on the integration of official healthcare, socioeconomic and territorial information to analyze the prevalence of diseases from the DH approach. Our findings encourage the incorporation of Geographical Information Systems (GIS) to support decision-making (Wang, 2020). In addition to broad patterns and relationships, GIS-based analyses offer interesting capabilities. An illustrative example is the possible underdiagnosis of depression in the elderly mentioned above. If such underdiagnosis is to be confirmed in a population group or in a given area, actions can be developed to this end.

It should also be mentioned the limitations of the study derived from the databases used. In mental diseases, the process of diagnosis, registration and treatment is subject to greater subjectivity comparted to the non-mental ones. The health inventory protocol of Aragon contains specific codes for mental pathologies that allow their unequivocal compilation (e.g., depressive disorder, anxiety disorder). However, the clinical tools for diagnosis, highly diverse and mainly based on clinical interviews, are subject to variability in medical practice (WHO, 2004; Muñoz and Jaramillo, 2015). Actions aimed at homogenizing the diagnostic tools may minimize the bias in the information derived from this variability, though they are not in place yet. There is also a temporal mismatch between prevalence data and the socioeconomic indicators, referred to 2011 as reported in the Spanish Population and Housing Census. Despite offering an adequate spatial coverage, their frequency and update timing hinders the multitemporal analysis.

Likewise, the suite of selected indicators was able to unravel broad relationships but might be lacking “thematic sensitivity,” holding limited capability to provide further insights. It would be of great interest to incorporate in future studies variables specifically related with structural processes that take place on a regional scale but with profound effects on DH manifestations at the local and the individual level. For instance, covariates linked to economic recession, prevailing situation in Spain at the beginning of the study period. These processes impact the labor and economic environments, leading to situations of precarious, temporary employment or unemployment; to migrant workers, to situations of lack of funds that ultimately result in sadness, despair, and isolation. This information related to individual factors is recorded by health administrations in patients’ medical records. Consequently, the identification of cause-effect relationships between structural and individual factors would lead to future studies, preferably of the observational type. In the case of the ecological type, a greater level of spatial and temporal disaggregation of the data will be sought, which will require joint work and continuous feedback between the scientific community and the public administrations.

## Conclusion

The interaction between depression prevalence and HD should not be reduced to the socioeconomic environment. There are other factors such as territorial development and organizational aspects of the health system that can support the explanation of the spatial distribution of depression prevalence from the DH approach. Public administrations offer information that, when integrated with that generated in the health system, can be used for research and decision-making aimed at reducing social and health inequalities. This demonstration case is focused on the prevalence of depression, one of the most prevalent and complex diseases in diagnosis and treatment in primary care. However, our approach can be easily adapted to other diseases and regions elsewhere.

## Data Availability Statement

The data analyzed in this study is subject to the following licenses/restrictions: Data on the prevalence of depression have been provided by the Research Ethics Committee of the Autonomous Community of Aragón (BIGAN database). Data on HD have been collected from official and public sources of the Government of Aragon: (i) https://idearagon.aragon.es/atlas/# (ii) https://www.aragon.es/-/indice-sintetico-desarrollo-territorial. Requests to access these datasets should be directed to CB-M, cbentue@unizar.es.

## Ethics Statement

The studies involving human participants were reviewed and approved by the Research Ethics Committee of the Autonomous Community of Aragon: CEICA. The ethics committee waived the requirement of written informed consent for participation.

## Author Contributions

CB-M: conceptualization, methodology, investigation, data curation, writing – original draft, and visualization. MR: conceptualization, methodology, investigation, software, validation, formal analysis, writing – review and editing, and supervision. RG-FL and JL: conceptualization, and writing – review and editing. MZ-A: conceptualization, methodology, investigation, resources, writing – review and editing, supervision, project administration, and funding acquisition. All authors contributed to the article and approved the submitted version.

## Conflict of Interest

The authors declare that the research was conducted in the absence of any commercial or financial relationships that could be construed as a potential conflict of interest.

## Publisher’s Note

All claims expressed in this article are solely those of the authors and do not necessarily represent those of their affiliated organizations, or those of the publisher, the editors and the reviewers. Any product that may be evaluated in this article, or claim that may be made by its manufacturer, is not guaranteed or endorsed by the publisher.
